# Detection of prognostic methylation markers by methylC-capture sequencing in acute myeloid leukemia

**DOI:** 10.18632/oncotarget.22789

**Published:** 2017-11-30

**Authors:** Yan Li, Hongmei Zhao, Qingyu Xu, Na Lv, Yu Jing, Lili Wang, Xiaowen Wang, Jing Guo, Lei Zhou, Jing Liu, Guofeng Chen, Chongjian Chen, Yonghui Li, Li Yu

**Affiliations:** ^1^ Department of Hematology and BMT Center, Chinese PLA General Hospital, Beijing 100853, China; ^2^ Department of Hematology, Hainan Branch of Chinese PLA General Hospital, Sanya 572013, China; ^3^ Annoroad Gene Technology Co. Ltd., Beijing 100176, China; ^4^ Medical School of Nankai University, Tianjin 300071, China; ^5^ Department of Hematology, General Hospital of Shenzhen University, Shenzhen 518060, China

**Keywords:** acute myeloid leukemia, next generation sequencing, MCC-Seq, DNA methylation, prognostic markers

## Abstract

Clinical and genetic features incompletely predict outcome in acute myeloid leukemia (AML). The value of clinical methylation assays for prognostic markers has not been extensively explored. We assess the prognostic implications of methylC-capture sequencing (MCC-Seq) in patients with de novo AML by integrating DNA methylation and genetic risk stratification. MCC-Seq assessed DNA methylation level in 44 samples. The differentially methylated regions associated with prognostic genetic information were identified. The selected prognostic DNA methylation markers were independently validated in two sets. MCC-Seq exhibited good performance in AML patients. A panel of 12 differentially methylated genes was identified with promoter hyper-differentially methylated regions associated with the outcome. Compared with a low M-value, a high M-value was associated with failure to achieve complete remission (*p* = 0.024), increased hazard for disease-free survival in the study set (*p* = 0.039) and poor overall survival in The Cancer Genome Atlas set (*p* = 0.038). Hematopoietic stem cell transplantation and survival outcomes were not adversely affected by a high M-value (*p* = 0.271). Our study establishes that MCC-Seq is a stable, reproducible, and cost-effective methylation assay in AML. A 12-gene M-value encompassing epigenetic and genetic prognostic information represented a valid prognostic marker for patients with AML.

## INTRODUCTION

Acute myeloid leukemia (AML) is a clonal disorder of myeloid hematopoiesis and a predominantly fatal hematopoietic malignancy with high heterogeneity [1]. Genetic heterogeneity has been appreciated since early karyotyping studies; somatic mutations have shown a comprehensive landscape of AML and contributed to disease classification and prognostic stratification [1–3]. However, a precise prognosis for nearly 50% of AML cases with a normal karyotype and patients with no mutations is still difficult to achieve [4]. DNA methylation, as the core and most widely studied epigenetic modification, is altered in numerous cancers and often correlates with clinically relevant information (i.e., subtypes, prognosis, and drug response) [5]. Indeed, aberrant DNA methylation is a common theme and a hallmark of AML [6]. Recent studies on genome-wide DNA methylation have emphasized the importance of dysregulated methylation profile in AML from biological and clinical views [7–11]. Aberrant DNA methylation has also been found suitable as a prognostic biomarker [7, 8, 10, 12–14]. However, the methylation techniques employed in these studies are difficult to apply in routine clinical practice [15].

Numerous DNA methylation detection techniques have thus far been developed, including those for genome scale and target enrichment methylation [15]. Currently, bisulfite treatment-based methylation microarray and next-generation sequencing (NGS) are commonly used for base resolution DNA methylomes, such as Illumina Human Methylation 450/850 BeadChip array (Illumina 450K/850K array) [16–18], whole-genome bisulfite sequencing (WGBS) [19], reduced representation bisulfite sequencing (RRBS) [20], Agilent SureSelect Human Methyl-Seq (www.genomics.agilent.com) [21], and NimbleGen SeqCap Epi CpGiant (www.nimblegen.com) [22]. With high robustness and accuracy, DNA methylation analysis based on an NGS platform has been confirmed to be feasible and reliable in clinical diagnosis and precision medicine, particularly for highly heterogeneous diseases such as AML [23]. However, only ∼20% or less of cytosine–phosphate–guanine (CpGs) are variable across individuals or tissues [24]. WGBS is inefficient for large-scale population studies because it entails high costs and requires in-depth sequencing capacity to achieve sufficient coverage. Meanwhile, RRBS is limited to the restriction enzyme cutting site in a fixed region, which can potentially result in loss of data with the lowest genome coverage [15]. Agilent SureSelect allows only single-strand capture of smaller target regions and requires larger amounts of input DNA, rendering it unsuitable for comprehensive genotype profiling [15]. Thus, alternative approaches to regulatory active functional methylome should be developed for comprehensive yet cost-effective identification of biologically and clinically relevant CpGs associated with complex diseases [25]. MethylC-capture sequencing (MCC-Seq) is an NGS capture approach that interrogates functional methylomes based on the NimbleGen SeqCap Epi CpGiant system with a unique design and long probes [26]. The technique provides comparable accuracy to alternative approaches but enables more efficient cataloguing of functional and disease-relevant methylation variants for large-scale epigenome-wide methylation studies [25, 26].

The current study presents MCC-Seq for the detection of prognostic methylation markers in AML and proposes a panel of 12 different functional DNA methylation genes.

## RESULTS

### MCC-Seq and study control

An outline of MCC-Seq is presented (Supplementary Figure 1). The sequence statistics obtained from 44 samples are summarized in Supplementary Table 1. We targeted more than 5Mb CpG sites of sequence covering genome-CpGs-scale with a total of 240,513 regions and a total size of 80Mb. The average percentages of CpG sites with coverage depths of no less than 1×, 5×, 10×, and 20× were 92.73%, 80.32%, 67.20%, and 43.81%, respectively. All 44 DNA samples for MCC-Seq (in excess of 30×) yielded 430Gb of sequence data. The converted rate for all samples exceeded 99.5%. A total of 58,147,036 (range: 40,663,694–79,302,058) clean reads on the average, were generated with an average of 95.03% (range: 92.65%–98.05%) clean Q30 base rate. Total mapping efficiency was 92.90 % (range: 80.52%–96.40%) and the average percentage of clean reads that mapped within the target CpGs was 72.32% (range: 38.54%–85.31%).

We further performed a sample-based validation of MCC-Seq. Two single bone marrow samples were obtained from randomly selected patients with AML in relapse (C22 and C23) and then prepared in replicate experiments (S22-Rep1 and S22-Rep2; S23-Rep1 and S23-Rep2). The effects of technical variability on methylation profiles were assessed by comparing the results of the replicates with independent captures and different degrees of multiplexing (≥ 1×, 5×, 10×, 20×) (Supplementary Materials). The results indicated highly concordant methylation calls for overlapping CpGs between S22-Rep1 and S22-Rep2, and the correlation improved with increasing read depth cutoffs (R = 0.959, 0.971, 0.978, 0.985 for the cutoffs of 1×, 5×, 10×, 20×), similar to S23-Rep1 and S23-Rep2 (Supplementary Figure 2).

In all subsequent population-based analyses in 37 samples with sequence depths ≥ 5×, a total of 5,068,466 CpGs were yielded for further consideration with an average sequence depth of 23.6× and a minimum of 5× (Supplementary Figure 3). Approximately 43.9% of the captured CpGs showed a hypomethylated pattern (< 20% methylation) and 48.5% exhibited hemi- to hypermethylated pattern (> 50% methylation) in the 21 samples with de novo AML (Supplementary Figure 4). For subsequent DMR analysis, a ≥ 10× coverage was required by removing those with below 10× coverage of sites over the 21 samples with de novo AML for distribution across all CpGs.

### Correlation between DNA methylation and clinical features

**Figure 1:**
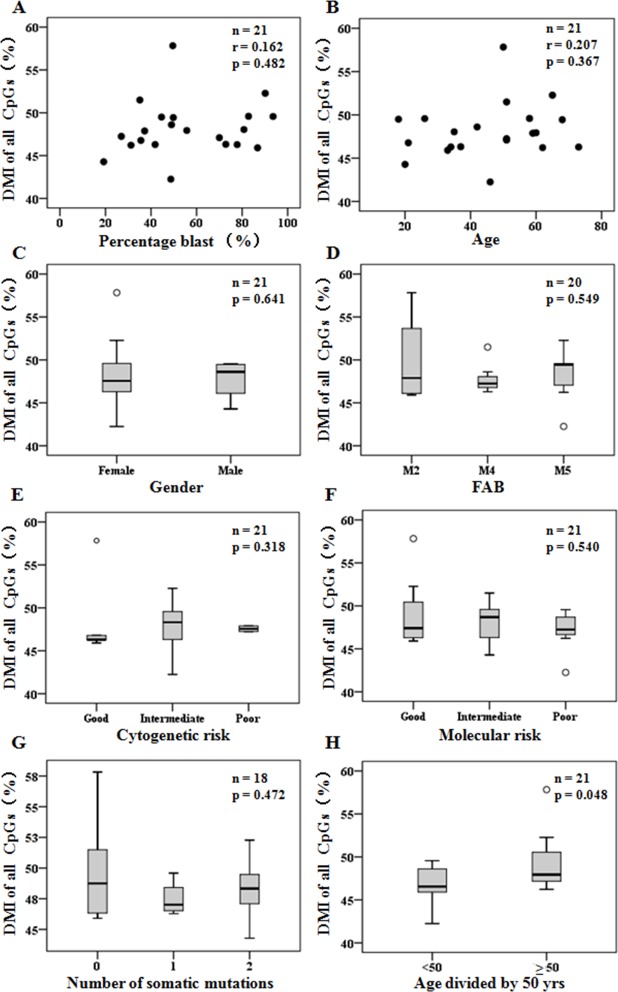
DMI of genome-wide captured CpGs detected in diagnosis were independent of clinical features **(A-B)** Scatter plots of DMI (%) compared to patient percentage blast of samples (A) and ages (B). **(C-H)** Box plots of DMI (%) grouped by patient gender (C), FAB disease classification (D), cytogenetic risk status (E), molecular risk status (F), number of somatic mutations (G), and age divided by 50 years old (H). Pearson correlation was used to determine r, and Student's *t* test or one-way ANOVA was used for mean tests.

We correlated some clinical features with the indicator of the DNA methylation level (DMI) (Supplementary Table 2) of genome-wide captured CpGs to determine whether a specific factor was associated with DMI in the 21 patients with de novo AML. Notably, the DMI was independent of the blast percentage of samples, age, gender, disease French–American–British (FAB) subtype, cytogenetic risk, molecular risk, and abundance of somatic mutations. Meanwhile, elder patients burdened a significantly higher DMI (≥ 50 y *vs.* < 50 y, 49.39% ± 3.43% *vs.* 46.75% ± 2.31%, *p* = 0.048) (Figure 1). To further assess associations between DMI and blast percentage of samples, we prepared DNA samples derived from sorted and concentration graded bone marrow with blast percentages of 70%, 80%, 90% and 100% of patient C21 (Supplementary Materials). We found a high correlation between each percentage of blast samples (R ≥ 0.95) with different read depth cutoffs (Supplementary Figure 5).

### Promoters have major different functional DNA methylation signatures

**Figure 2:**
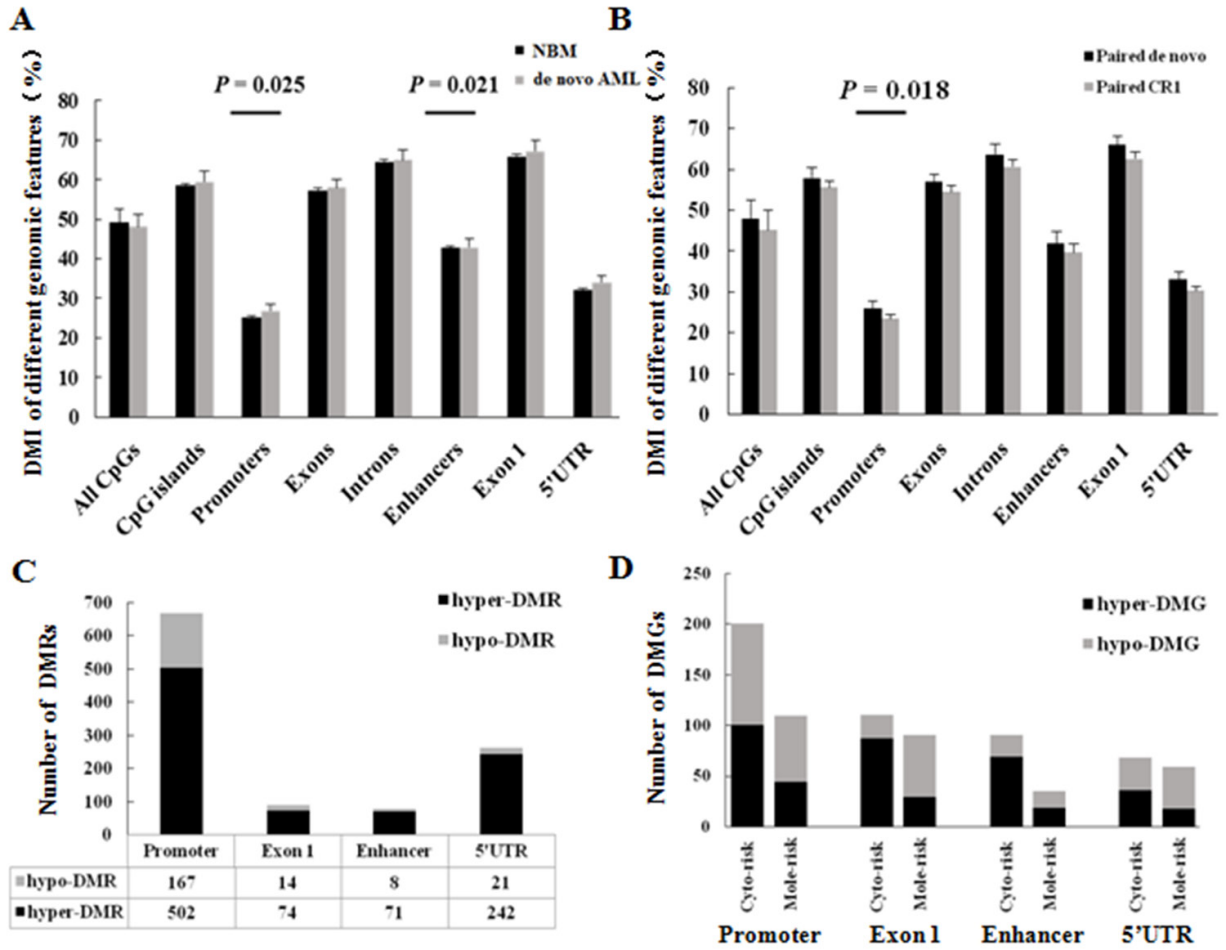
Promoters have major different functional DNA methylation signatures **(A)** DMI of different genomic features compared between de novo AML and NBM samples. Student's *t* test was used for mean tests. **(B)** DMI of different genomic features compared between 8 paired de novo AML and complete remission 1 (CR1) samples. **(C)** DMRs in functional elements between de novo AML and NBM samples. **(D)** DMGs in promoters compared among prognostic stratifications. Cyto-risk, Cytogenetics risk; Mole-risk, Molecular risk.

As MCC-Seq has a genome-wide scale coverage of CpGs, the DNA methylation signature of different genomic features (i.e. all CpGs, CpG islands, promoters, exons, exon 1, introns, enhancers, 5’untranslated region (5’UTR), etc.) (Supplementary Table 2) was compared among different clinical groups. Comparison of the DMI of these genomic features between de novo AML and normal bone marrow (NBM) indicated that only the DMI in promoters and enhancers were significantly higher in AML (*p* = 0.025 and *p* = 0.021, respectively) (Figure 2A). Furthermore, a significant decrease in DMI in promoters (*p* = 0.018) was observed but not in enhancers (*p* = 0.145) by comparing the results from 8 paired samples (complete remission 1 (CR1) samples *vs.* diagnosis samples) (Figure 2B). Comparison of results from 3 other paired samples indicated that the DMI in promoters was similar between diagnosis and relapsed samples (*p* = 0.305). These results could indicate that the DNA methylation signature in promoters was representative in AML and associated with clinical response.

We compared the number of differentially methylated regions (DMRs) in mainly functional elements (promoter, exon 1, enhancer, 5’UTR) between de novo AML samples and NBM; the DMRs had the greatest number of promoters (60.9%, 669/1099, *p* < 0.001), which mainly consisted of hyper-DMRs (75.0%, 502/669, *p* < 0.001) (Figure 2C). We also compared the DMRs among different cytogenetic risk groups and molecular risk groups within AMLs, then annotated genes that refered to these DMRs in functional elements (mainly in the promoter and enhancer regions)—that is, differentially methylated genes (DMGs) [22]. The results demonstrated that most DMGs were associated in promoters (*p* < 0.001) (Figure 2D, Supplementary Table 3). Thus, these results indicated that promoters have major different functional DNA methylation signatures, which were associated with established genetic prognostic stratifications. We subsequently explored prognostic methylation markers according to the DNA methylation signatures in promoters.

### Differentially methylated genes associated with established prognostic stratification

**Figure 3:**
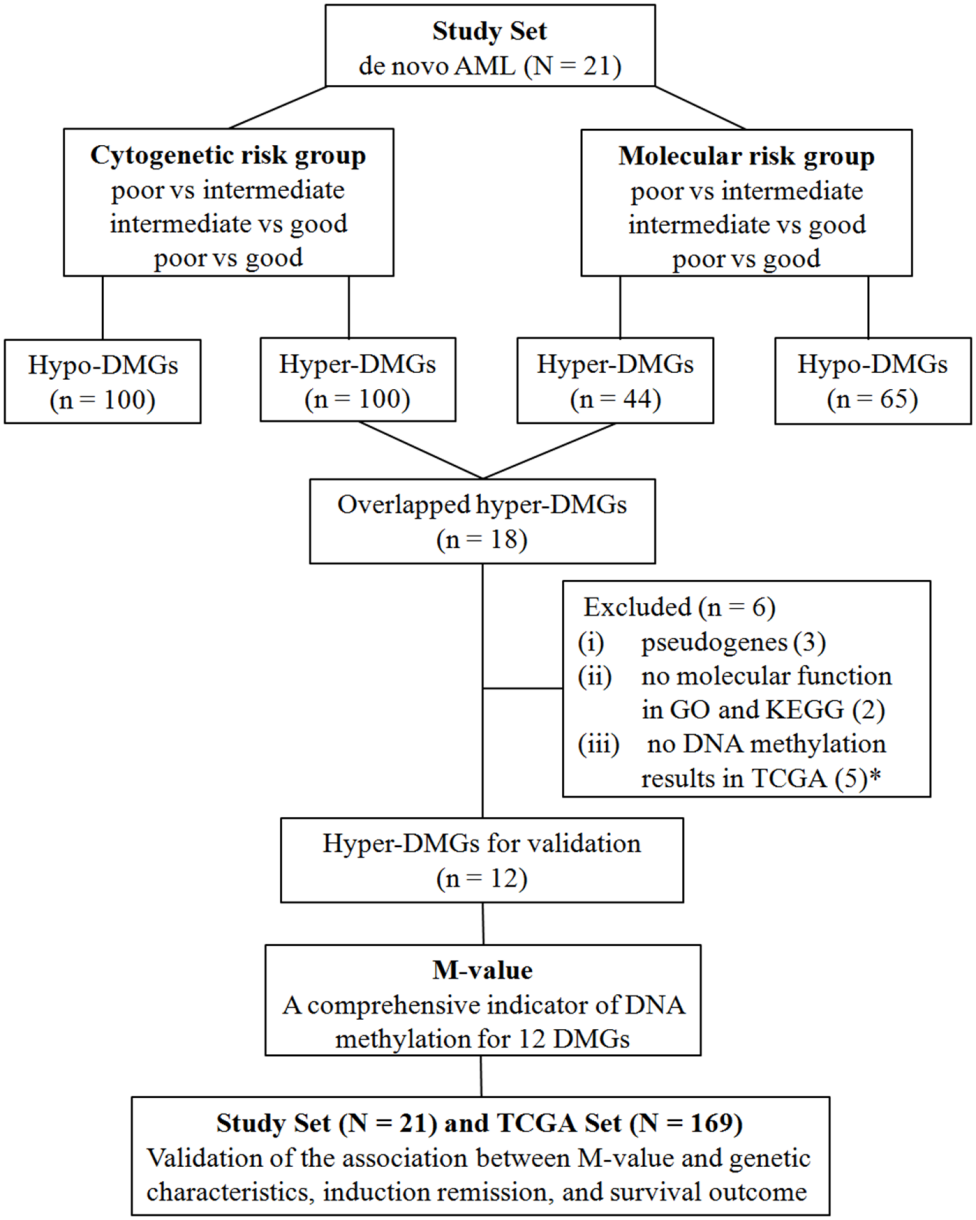
Diagram of the generation and validation of differentially methylated genes according to the DMRs in promoter N, number of patients; n, number of genes; ^*^ , 4 genes were doubly counted, 2 of which were pseudogenes and the other 2 were without molecular functions in GO and KEGG analysis.

A diagram illustrating the generation and validation of annotated DMGs according to the DMRs in promoter regions is summarized (Figure 3). To identify unfavorable genes with hyper DNA methylation and develop a molecular risk panel that incorporated both epigenetic and genetic prognostic information, we compared the DMRs in promoters among the different cytogenetic risk groups and molecular risk groups of 21 patients with de novo AML patients, both poor *vs.* intermediate, intermediate vs. good and poor *vs.* good subgroups. A total of 100 hyper-DMGs were generated from the comparison of cytogenetic risks. Meanwhile, 44 hyper-DMGs were generated from the comparison of molecular risks (Supplementary Table 3). Subsequently, 18 hyper-DMGs were obtained by overlapping the 100 and 44 hyper-DMGs, both of which were associated with higher cytogenetic and molecular risks (Table 1).

**Table 1:** 18 hyper-DMGs associated with higher cytogenetic and molecular risks

Gene symbol	Full name	Chr. location	Role in cancer	ID in NCBI gene database
BARD1	BRCA1 associated RING domain 1	2q35	Down-regulation in MDS with progression to AML, tumor suppressor genes [27]	580
BCL9L	B-cell CLL/lymphoma 9-like	11q23.3	Down-regulation associated with tumor cell migration in ovarian cancer [28]	283149
CLEC11A	C-type lectin domain family 11 member A	19q13.33	Hyper-methylation in pancreatic cancer [29]; Associated with leukemia cell proliferation [30]	6320
DEFB1	defensing beta 1	8p23.1	DNA methylation-mediated down-regulation in prostate cancer [31]; tumor suppressor genes [32]	1672
FOXD2	forkhead box D2	1p33	DNA methylation-mediated down-regulation in colorectal cancer [33]; tumor suppressor genes [34]	2306
GUCY1B2^a^	guanylate cyclase 1 soluble subunit beta 2 (pseudogene)	13q14.3	Pseudogene	2974
HNRNPA1P33^a^	heterogeneous nuclear ribonucleoprotein A1 pseudogene 33	10q11.22	Pseudogene	728643
IGF1	insulin like growth factor 1	12q23.2	Hyper-methylation involved in solid tumor [35]	3479
IL18	interleukin 18	11q23.1	Dual role involved in solid tumor [36, 37]	360
ITIH1	inter-alpha-trypsin inhibitor heavy chain 1	3p21.1	Down-regulation involved in solid tumor [38]	3697
LSP1	lymphocyte-specific protein 1	11p15.5	Regulated by DNA methylation [39]; Low expression in breast cancer [40]	4046
MIR3150B^a^	microRNA 3150b	8q22.1	High expression in breast tumor [41]	100500907
MIR4638^a^	microRNA 4638	5q35.3	High expression in breast tumor [41]	100616342
P2RX6	purinergic receptor P2X 6	22q11.21	Regulated by p53, role in cancer unknown [42]	9127
PLEC^a^	plectin	8q24.3	With genetic and epigenetic alterations in AML [43]	5339
RNASE3	ribonuclease A family member 3	14q11.2	Low expression in pancreatic cancer [44]	6037
TUBA3FP^a^	tubulin alpha 3f pseudogene	22q11.21	Pseudogene	113691
TUBGCP2	tubulin gamma complex associated protein 2	10q26.3	High expression in AML [45]	10844

^a^ These 6 genes were excluded for clinical validation.

Additional details, including the role of these 18 genes in cancer are summarized in Table 1. Except for 3 pseudogenes (*GUCY1B2*, *HNRNPA1P33*, and *TUBA3FP*), all of the remaining 15 genes showed the most involvement as tumor suppressor genes in distinct carcinomas, and four genes (*TUBGCP2*, *PLEC*, *CLEC11A*, and *BARD1*) were associated with AML. In addition, 7 genes (*PLEC*, *LSP1*, *IL18*, *IGF1*, *FOXD2*, D*EFB1*, and *CLEC11A*) were reportedly regulated by DNA methylation. Gene Ontology (GO) and Kyoto Encyclopedia of Genes and Genomes (KEGG) pathways analysis were performed for the remaining 15 genes and 2 genes (*MIR3150B* and *MIR4638*) had no molecular function. We downloaded the DNA methylation datasets of AML for the 18 genes from The Cancer Genome Atlas (TCGA) data portal; however, no DNA methylation results on 5 genes (*HNRNPA1P33, TUBA3F, MIR3150B, MIR4638, and PLEC*) were available. Six genes were excluded due to pseudogenes, no molecular function, or no results in TCGA. A panel of 12 functional DMGs (*BARD1*, *BCL9L*, *CLEC11A*, *DEFB1*, *FOXD2*, *IGF1*, *IL18*, *ITIH1*, *LSP1*, *P2RX6*, *RNASE3*, and *TUBGCP2*) associated with established genetic prognostic stratification were generated, and the prognostic significance was evaluated (Figure 3).

### Validation of 12-DMG panel for clinical implications

We calculated the DNA methylation level of individual patients, based on the 12 DMGs. The M-value, which was the mean DMI of 12 DMGs for individual patients, was obtained. The clinical impact of M-value was then tested in 21 patients with AML in our study and validated in 169 patients with AML in the TCGA study.

#### M-value is not associated with patient characteristics

We correlated some clinical features with the M-value of the 12 DMGs to determine whether a specific factor was associated with the M-value both in the study set and TCGA set. Similarly, the M-value was independent of the age, gender, and blast percentage of the samples, similar to the DMI in all captured CpGs. However, patients with a higher cell differentiation subtype of FAB in the TCGA set burdened a higher M-value (*p* < 0.001) (Supplementary Figure 6).

#### M-value is significantly associated with AML genetic characteristics

**Figure 4:**
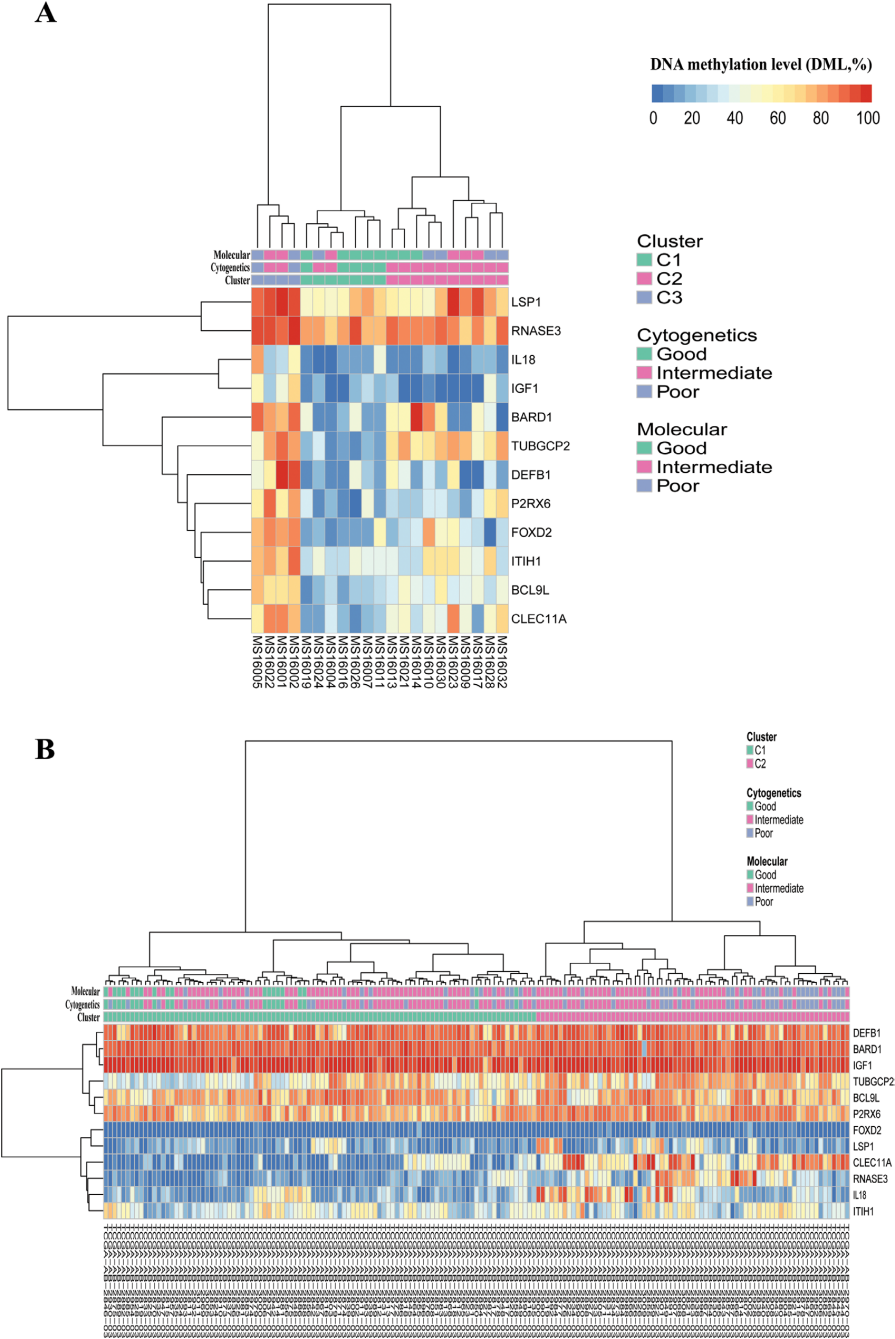
Hierarchical clustering of the study set (**A**, n = 21) and the TCGA set (**B**, n = 169) according to their methylation profiles of the 12 DMGs grouped by clusters, cytogenetics risk stratifications and molecular risk stratifications, respectively.

Given the relationship of genetic characteristics and outcome in AML, we assessed the association of the M-value with AML genetic characteristics. In both the study set and the TCGA set, the M-value was shown to significantly improve with increasing cytogenetic and molecular risk stratifications (Table 2, Supplementary Figure 7). Moreover, unsupervised analysis using hierarchical clustering suggested that the patients in the study could be segregated into 3 groups (cluster 1, n = 7; cluster 2, n = 10; cluster 3, n = 4) according to the methylation profiles of these 12 DMGs (Figure 4A). The M-values of these 3 clusters were significantly different (26.59% ± 4.36% vs. 41.08% ± 4.72% vs. 68.47% ± 4.33%, *p* = 0.001). Notably, all 5 patients with good-risk cytogenetics were in cluster 1 with a low M-value; 2 patients with poor-risk cytogenetic were in cluster 3 with a high M-value; and 10 of 14 patients with intermediate-risk cytogenetics were in cluster 2 with an intermediate M-value. Four patients with cytogenetics intermediate-risk group (S01, S03, S15, S17-1) were assigned to low M-value cluster 1 (S03, S17-1) and high M-value cluster 3 (S01, S15). Hierarchical clustering of 169 patients with AML from the TCGA data portal showed that patients could be segregated into 2 groups (cluster 1, n = 98; cluster 2, n = 71) according to the methylation profiles of these 12 DMGs (Figure 4B). The M-value of these 2 clusters were significantly different (47.22% ± 4.32% vs. 59.11% ± 5.42%, *p* < 0.001). Notably, 79 of 108 patients with intermediate-risk cytogenetic were assigned to cluster 1 with a low M-value, whereas 29 patients were assigned to cluster 2 with a high M-value, similarly to the case with molecular intermediate risk (79 to cluster 1 and 22 to cluster 2). These results suggested a high consistency between the DNA methylation profiles of 12 DMGs and the genetic signature for AML prognosis. Interestingly, some patients with intermediate-risk cytogenetics group could be distinguished by the M-value, which could further improve the prognosis stratification.

**Table 2:** Correlation between M-value and genetic risk stratifications

	Study set (n = 21)	TCGA set (n = 169)
**Cytogenetic risk**		
Good	27.89% ± 4.42% (n = 5)	47.01% ± 4.59% (n = 19)
Intermediate	42.31% ± 13.01% (n = 14)	51.96% ± 7.28% (n = 108)
Poor	69.96% ± 6.95% (n = 2)	55.23% ± 8.17% (n = 42)
*P* value	0.001	0.000
**Molecular risk**		
Good	32.01% ± 6.69% (n = 8)	47.01% ± 4.59% (n = 19)
Intermediate	45.61% ± 18.76 (n = 6)	52.19% ± 7.41% (n = 101)
Poor	48.84% ± 16.38 (n = 7)	54.29% ± 8.03% (n = 49)
*P* value	0.047	0.002

#### M-value is associated with AML induction remission

The mean M-value for patients in CR was lower than that for patients with no response (NR) (37.42% ± 15.79% *vs.* 49.69% ± 12.51%, *p* = 0.09). To further assess the association between the M-value and CR, we divided the study set of 21 patients with AML to the low M-value group (n = 11) and the high M-value group (n = 10) according to the median of the M-value (Supplementary Table 4). Approximately, 90.9% (10/11) of the patients achieved CR in the low M-value group, which was significantly higher than that in the high M-value group 40.0%, 4/10; *p* = 0.024). Moreover, 5 of 6 (83.3%) intermediate-risk AML (IR-AML) with a low M-value achieved CR, whereas only 3 of 8 (37.5%) IR-AML with a high M-value achieved CR. We also found that the CR rate remained the same regardless of the regimen received by the patients—that is, the standard “7+3” regimen or the “decitabine + cytarabine + aclarubicin + granulocyte colony-stimulating factor” (DCAG) regimen (Supplementary Table 4). These results indicated that patients with a low M-value were more likely to achieve CR in both total AML and IR-AML, independent of the induction regimen. This finding could not be verified in the TCGA set because of lack of information regarding induction remission response.

#### M-value is associated with survival

**Figure 5:**
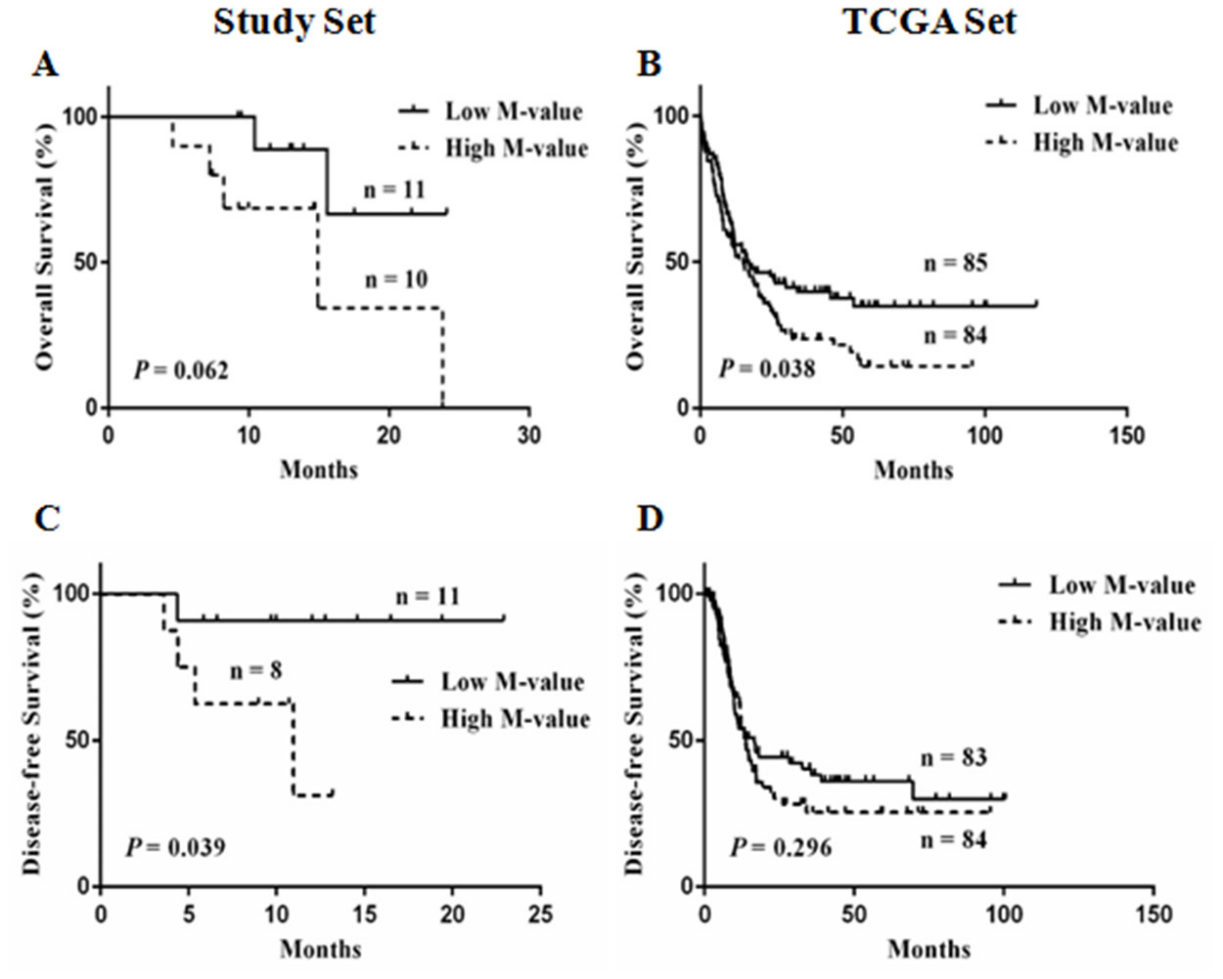
Kaplan-Meier curves for low and high M-value groups **(A, C)** overall survival (OS) and disease-free survival (DFS) of the study set; **(B, D)** OS and DFS of the TCGA set.

The relationship between M-value and survival was also examined both in the study set and the TCGA set (Figure 5). The patients were divided into the low M-value group and the high M-value group, as determined by the median of the M-value in each data set (Supplementary Table 5). First, the median overall survival (OS) / disease-free survival (DFS) and 1-year cumulative OS/DFS of the 21 patients with AML were 23.8 months/not defined and 78.9%/69.1%, respectively (Table 3). A high M-value was associated with increased hazard for DFS alone (HR: 6.83, 95%CI: 1.07–40.28) (Figure 5C). The values obtained for the low M-value group and the high M-value group were as follows: median OS, not defined and 14.93 months (*p* = 0.062); DFS, not defined and 10.97 months (*p* = 0.039); 1-year cumulative OS, 88.9% and 68.6% (*p* = 0.145); and DFS, 90.9% and 30.0% (*p* < 0.001), respectively (Figure 5A, 5C).

**Table 3:** Patient and sample characteristics

Patient characteristics	N = 21
Age (y)	45.71 ± 16.50
Male sex: no. (%)	7 (33.3)
**AML FAB subtype: no. (%)**	
AML with maturation: M2	4 (19.0)
Acute myelomonocytic leukemia: M4	9 (42.9)
Acute monoblastic or monocytic leukemia: M5	7 (33.3)
Acute erythroid leukemia: M6	1 (4.8)
**AML WHO subtype: no. (%)**	
AML, NOS	8 (38.1)
AML with t(8;21)(q22;q22.1)	3 (14.3)
AML with MDS-related changes	2 (9.5)
AML with biallelic mutations of *CEBPA*	4 (19.1)
AML with CBFB-MYH11	2 (9.5)
AML with mutated *NPM1*	2 (9.5)
Bone marrow blasts at diagnosis:%	64.46 ± 19.98
**Normal cytogenetic profile: no. (%)**	9 (42.9)
Whitecell count at diagnosis: per mm^3^	
Mean	21,822 ± 34,371
Median (range)	11,390 (590, 137,630)
**Cytogenetic risk group: no. (%)**	
Good	5 (23.8)
Intermediate	14 (66.7)
Poor	2 (9.5)
**Molecular risk group: no. (%)**	
Good	8 (38.1)
Intermediate	6 (28.6)
Poor	7 (33.3)
**Remission induction: no. (%)**	
7+3^*^	9 (42.9)
DCAG^#^	12 (57.1)
**Response: no. (%)**	
Complete remission (CR)	14 (66.7)
No response (NR)	7 (33.3)
Median follow-up (OS/DFS)	12.9 Months/ 10.0 Months
1-year Cumulative OS	78.9% ± 9.6%
1-year Cumulative DFS	69.1 ± 12.3%
Median OS	23.8 Months
Median DFS	/
**Sample characteristics**	**N = 44**
NBM	N = 5
AML samples	N = 39
De novo	21
Paired complete remission (cycle 1)	8
Paired relapsed	3
Concentration gradients	3
Reduplicate	4

^*^ “7+3”, standard induction regimens based on a backbone of cytarabine plus an anthracycline, with details according to the NCCN guideline for AML.

^#^ DCAG, decitabine 20mg/m^2^ d1-5, cytarabine 10mg/m^2^ q12h d1-5, aclarubicin 20mg d1, 3, 5, G-CSF 300μg/d until recovery from neutropenia.

FAB, French–American–British; WHO, World Health Organization; OS, overall survival; DFS, disease-free survival; NBM, normal bone marrow.

In the TCGA set, the high M-value group obtained a significantly poor OS than that of the low M-value group (median OS: 15.1 months *vs.* 16.4 months; HR: 1.491, 95%CI: 1.043-2.151, *p* = 0.038) (Figure 5B). A trend in unfavorable prognosis for DFS without significant difference was observed in the high M-value group (median DFS: 13.8 months *vs.* 16.6 months; HR: 1.251, 95%CI: 0.822-1.920, *p* = 0.296) (Figure 5D). In addition, the high M-value group showed significantly lower 2-year OS and DFS (OS: 35.9% *vs.* 45.9%, *p* = 0.001; DFS: 30.2% *vs.* 44.2%, *p* < 0.001). The M-value based risk was meaningful for OS (*p* = 0.038, Figure 5B), and treatment with hematopoietic stem cell transplantation (HSCT) significantly improved OS (HSCT *vs.* no HSCT, median OS: 27 months vs. 7.7 months; HR: 0.4846, 95%CI: 0.3214-0.6738, *p* < 0.0001, Supplementary Figure 8A); thus, we assessed whether HSCT alters the M-value based risk. The results indicated that HSCT and survival outcomes were not adversely affected by the high M-value (*p* = 0.271, Supplementary Figure 8B), which suggested that the adverse prognostic effect of a high M-value could be mitigated by HSCT.

## DISCUSSION

In this study, genome-wide MCC-Seq was primarily used to detect prognostic DNA methylation markers in patients with AML. The sequencing profile showed a good converted rate (> 99.5%), clean Q30 base rate (95.03%), mapping efficiency (92.90%), and a high concordance of replicate experiments. These results indicated that MCC-Seq is stable, reproducible and suitable for the analysis of bone marrow samples. By a series of comparative screening, a panel of 12 DMGs was identified with promoter hyper-DMRs associated with the outcome. A high M-value was associated with failure to achieve CR and poor survival, and its adverse prognostic effect could be mitigated by HSCT.

MCC-Seq is a NGS capture approach with the quantitative detection of DNA methylationn level (defined as DMI). To promote the clinical application of MCC-Seq in AML, we assessed the potential factors that could influence the DMI, which was found to be not associated with patient characteristics (e.g., age, gender, blast percentage of samples, FAB classification, etc.). Independent of blast percentage, DMI was further confirmed by bone marrow grading with different blast percentages from the same sample. These results were consistent with previous studies despite the differences in AML cohort, methods of detection and analysis, genomic regions analyzed, and DNA methylation index [10, 13, 46, 47]. Thus, BM blasts were regarded as a mere index of disease burden that, exerted no influence on methylome analysis. The reason was unclear and could suggest that DNA methylation assays reflect aberrant methylation in both blasts and more differentiated myeloid cells derived from leukemic precursors [13, 46]. The independence and stability of DNA methylation analysis renders it suitable as a prognostic biomarker in AML.

DNA methylation is important for gene silencing via the hypermethylation of CpG islands in promoter regions [48]. The DNA promoter regions of critical tumor suppressor genes are inactivated via hypermethylation, which seems to significantly influence the pathogenesis and prognosis of AML [49, 50]. Recent DNA methylome studies demonstrated that multi-locus DNA methylation assay in promoters can predict outcomes in de novo AML [7, 12–14]. Meanwhile, some studies revealed more diverse DNA methylation functions dependent on genomic location, particularly in enhancers [10, 24, 48, 51]. In the current study, with a genome-wide scale coverage of CpGs by MCC-Seq, the DNA methylation signature of different genomic features was evaluated to avoid regional bias. Consequently, promoters obtained a significantly higher DMI in de novo AML (*p* = 0.025) and was significantly lower in CR1 (*p* = 0.018). The most number of DMRs and DMGs were distributed in promoters compared with other regions (*p* < 0.001). These results collectively indicated that promoters have major different functional DNA methylation signatures in AML, as demonstrated in previous and recent studies [6, 9, 10, 12, 47].

AML is a complex disease with genetic and epigenetic changes [3, 10]. However, classification and prognostication of the disease for AML patients have thus far been largely dependent on cytogenetic and genetic testing (recurrent somatic mutations), whereas epigenetic changes, including DNA methylation, have not been considered [1]. Marcucci *et al.* were the first to integrate genetic and epigenetic information for prognostication and treatment response prediction in AML [12]. They reported a gene expression score involving 7 oncogenes associated with somatic mutations and DNA methylation for a meaningful prognosis. The limitation was that the genes were derived from a cohort of older patients with cytogenetically normal AML (CN-AML) and only validated in CN-AML sets. The present study encompassed both genetic (cytogenetic risk and molecular risk) and epigenetic (DNA methylation) information from a new standpoint to develop a novel prognostic gene panel in all non AML-M3 subtypes. On the basis of cytogenetic and molecular risk stratification, we identified DMRs and DMGs to select hypermethylated genes in higher-risk stratification subgroups. To ensure the accuracy and reliability of these hyper-DMGs, 18 overlapping hyper-DMGs associated with both increased risk of cytogenetics and molecular stratification were obtained. A panel of 12 DMGs with a strict selection process was formed. Most of these genes acted as tumor suppressors in cancer and were regulated by DNA methylation (Table 1), which was consistent with our screening process (genes hypermethylated in the higher-risk group).

The prognostic value of these 12 DMGs was evaluated in the study set and the TCGA set. The M-value of the 12 DMGs was significantly associated with AML genetic characteristics. Consistent with previous studies, the M-value obtained in the present study not only predicted CR rates and DFS or OS duration in all patients with AML but also represented the prognostic value for patients in CR with IR-AML [10, 12–14]. Luskin *et al.* did not assess whether HSCT alters their DNA methylation-based risk because of insufficient power in their cohort [13]. In the current study, we investigated this issue by using the M-value-based risk in 81 patients with HSCT from the TCGA set. The results indicated that HSCT may mitigate the adverse prognostic effect of high M-value. High M-values were associated with failure to achieve CR and with poor OS or DFS; however, patients with high M-values could benefit from HSCT, which is clearly an essential area of future investigation.

In summary, genome-CpGs-scale detection of prognostic methylation markers by MCC-Seq is feasible and revealed an M-value for 12 genes that could be used as a valuable biomarker for risk stratification. The M-value is suitable for all patients with AML, particularly those without genetic and molecular markers. It is expected to be used as a biomarker to guide demethylation therapy. Patients predicted to have poor outcomes based on high M-value may benefit from more intensive post-remission treatment (e.g., HSCT) or enrollment in a clinical trial. None of these 12 genes were reported in other prognostic studies based on methylation status [7, 9, 10, 13, 14]. However, methodologic differences prevented a direct, meaningful comparison of our integrated genetic risk and different DNA methylation prognostic markers with previously reported results. Our conclusions are currently limited, given the small size of the cohort study. The prognostic value of the M-value has to be verified in future larger-scale studies.

## MATERIALS AND METHODS

### Ethics statement

The study protocol was approved by the author's institutional ethics committee, the Ethics Committee of the General Hospital of Chinese People's Liberation Army, and was conducted in accordance with the Declaration of Helsinki. Written informed consent was obtained from each participant prior to specimen collection.

### Patients and samples

A total of 21 patients with de novo AML, 2 patients with AML in relapse, and 5 healthy donors for related allogeneic HSCT who visited our hematology department between August 2014 and June 2016 were enrolled in the study. DNA samples from bone marrow and clinical information were collected (Supplementary Materials). A total of 35 samples from the bone marrow of 21 adult patients with AML (non AML-M3) were obtained. In addition, four replicate samples were obtained from another set of 2 randomly selected relapsed AML patients, and 5 samples from NBM were obtained. The characteristics of the patients with de novo AML and all samples are fully described in Table 3 and Supplementary Table 6. The diagnosis and prognosis of AML were based on World Health Organization 2016 classification and the AML guidelines of the National Comprehensive Cancer Network (NCCN; AML, Version 1.2017; http://www.nccn.org/). The 21 patients consisted of 14 women and 7 men with a median age of 50 y (range, 18-73 y). Median follow-up was 12.9 months (range, 4.6-24.1 months) for OS and 10.0 months (range, 3.6-22.9 months) for DFS.

### MCC-Seq protocol

A total of 44 DNA samples were used for MCC-Seq (Table 3). The concentration and integrity of DNA were detected by electrophoresis to confirm the quality. In MCC-Seq, a whole-genome methylation sequencing library is prepared with the qualified DNA samples, bisulfate-converted, and amplified, followed by a capture enriched for targeted bisulfite-converted DNA fragments. This process is achieved using the novel SeqCap Epi probe design platform developed by Roche NimbleGen. This platform enables the capture of double-stranded targets regardless of their methylated state via high-density tiling of probes [22, 25]. Each capture was sequenced on a single lane of the 125 bp paired-end Illumina HiSeq2500 System (Supplementary Materials).

### MCC-Seq methylation analysis

The glossaries used in this study are summarized in Supplementary Table 2. Raw sequence reads were filtered to remove adapter contamination and poor-quality reads. Clean sequences were first mapped to the human genome (build GRCh37) by using Bismark (v0.10.1; parameters: –pe, –bowtie2, –directional, –unmapped). Methylation calls were extracted after duplicate sequences had been excluded. DMRs were analyzed in R 3.1.0 by using the methylKit package. The minimum read coverage to call a methylation status for a base was set to 5. All off-target reads were removed. The methylation level at each site was determined by dividing the number of reads supporting methylation for that site by the total number of reads covering that site. CpGs were included in subsequent analyses if the number of sequence reads was 5 or greater. Data visualization and analysis were performed using Integrative Genomics Viewer, custom R, and Perl scripts (Supplementary Materials).

To ensure the reliability of the sequencing results without bias, both the technicians and bioinformatics analyst were blinded to the clinical information of the samples.

### Analyses of DNA methylation data from TCGA AML cohort

TCGA performed profiling using Illumina Infinium HumanMethylation450 BeadChip for 194 samples of 200 clinically annotated adult cases of de novo AML [3]. Clinical data and DNA methylation datasets for the AML cohort are publicly available through the TCGA data portal (https://tcga-data.nci.nih.gov/tcga/). The MCC-Seq platform targets the same set of genes as the 450K (99% of RefSeq genes) [15, 22]; thus, we included 169 patients with non AML-M3 with complete cytogenetic/molecular risk information and DNA methylation profiles into this study to validate the correlation of DNA methylation and clinical features (Supplementary Table 7).

### Statistical analyses

The demographics and characteristics were summarized using descriptive statistics. Student's *t* test or the Mann-Whitney U-test were used to compare continuous variables. Categorical variables were compared using the Fisher's exact test or Chi-square test. Outcome measures were assessed using Kaplan-Meier estimates in a univariate analysis. OS was defined as the time from diagnosis to death from any cause or last follow-up. DFS was defined as the time from CR to the date of relapse, death, or last follow-up. A two-sided p-value < 0.05 was considered statistically significant. All statistical analyses were performed with SPSS software version 19.0 (IBM Corp., Armonk, NY, USA), GraphPad Prism 6 (GraphPad Software Inc., San Diego, California, USA).

## SUPPLEMENTARY MATERIALS FIGURES AND TABLES










