# Supplementary material for: Detection of prognostic methylation markers by methylC-capture sequencing in acute myeloid leukemia
Source: Oncotarget. 2017 Nov 30;8(66):110444–59. doi: 10.18632/oncotarget.22789 (PMC5746395; doi:10.18632/oncotarget.22789)
Supplement: Supplementary file 1 [file oncotarget-08-110444-s001.pdf]

Detection of prognostic methylation markers by methylC-capture sequencing in acute myeloid leukemia

SUPPLEMENTARY MATERIALS

This information has been provided by the authors to give readers additional information about their work.

Patients and samples

A total of 21 patients with de novo AML, 2 patients with AML in relapse, and 5 healthy donors for related allogeneic hematopoietic stem cell transplantation (allo-HSCT) were enrolled in the study. All bone marrow DNA samples were randomly selected throughout more than 400 samples from the clinical specimen bank of our hospital to reflect a real life distribution of subtypes and status [1–3], except for the concentration gradients and replicate BM samples. Sample inventory, quality issues and complete clinical data also had to be considered in the selection process. The charts, electronic medicated records, and laboratory records (bone marrow morphology, immunology, chromosome karyotypes, molecular abnormalities, etc.) were reviewed by two trained hematologists. Data on demographic characteristics, diagnosis and treatment of AML, survival status, etc. were collected and recorded in the case report form. Finally, all the data were put into an Excel database and further evaluated by a third trained hematologist.

Concentration gradients samples

The concentration gradients samples were collected from a randomly selected de novo patients, numbered as C21 in this study. Ten-milliliter (2 tubes) ethylene diamine tetraacetic acid (EDTA)-anti coagulated fresh bone marrow samples were collected at diagnosis. Of which 2ml was used for identifying immunophenotype by flow cytometry (FCM) and the remaining was for extracting mononuclear cells. The antibody of CD117 was finally determined for labelling leukemia cells (70%) based on immunophenotype from FCM. Meanwhile, the mononuclear cells were equally divided into two samples: Sample 1 and 2. The Sample 2 was labelled with the CD117 antibody (Anti-Human CD117 (c-Kit) PE-Cyanine5), with flow cell sorting conducted to obtain 100% purify of leukemia cells by BD FACSAria (BD Biosciences, USA). Finally, after calculating and gradient mixture between Sample 1 and absolutely purified leukemia cells, we got Concentration gradients samples with different purity of cells: 70%, 80%, 90% and 100%, numbered as S21-Sor1, S21-Sor2, S21-Sor3 and S21-Sor4, which was then used for MCC-Seq to assess associations between DNA methylation level (DMI) and percentage blast of specimens.

Replicate samples

To assess the effects of technical variability of MCC-Seq in bone marrow DNA samples, another two single patients with AML in relapse (C22 and C23) were randomly selected. Five-milliliter ethylene diamine tetraacetic acid (EDTA)-anti coagulated fresh bone marrow samples were collected and mononuclear cells were extracted, respectively. Then the mononuclear cells were equally divided into two samples of each patient, therefore four replicate samples were obtained, numbered as S22-Rep1 and S22-Rep2, S22-Rep1 and S22-Rep2. Subsequently, DNA was extracted for MCC-Seq.

MCC-Seq protocol

MCC-Seq has been confirmed to be a comparable accuracy to alternative approaches but enables more efficient cataloguing of functional and disease-relevant epigenetic and genetic variants for large-scale EWAS by Allum F *et al.* [4]. The MCC-Seq performed on our platform has been showed a good performance (Supplementary Table 1 and Supplementary Figure 1).

More details in this study:

MCC-Seq library construction

The concentration and integrity of DNA were detected by electrophoresis to confirm the quality. Genomic DNA (500ng-1μg) was fragment into 200 bp peak sizes using the Covaris focused-ultrasonicator E210. Fragment size was controlled on a Bioanalyzer DNA 1000 Chip (Agilent) and the KAPA HTP Library Preparation Kit (KAPA Biosystems) was applied. End repair of the dsDNA which produces blunt-ended'5-phosphorylated fragments; then A-taling, during which dAMP is added to the 3-ends of blunt-ended dsDNA library fragments; Methylated-adapter ligation and clean-up steps were carried out as per KAPA Biosystems' recommendations. The cleaned-up ligation product was then analysed on a Bioanalyzer High Sensitivity DNA Chip (Agilent) and quantified by PicoGreen (Life Technologies).

Bisulfite conversion

The cleaned-up ligation products were bisulfite converted using the EZ DNA Methylation-Gold™ Kit (Zymo Research, Irvine, CA, USA) as described by the manufacturer. Bisulfite converted DNA was quantified using Qubit 2.0 (Life Technologies) and, based on quantity, amplified by 9-12 cycles of PCR using the Kapa Hifi Uracil + DNA polymerase (KAPA Biosystems), according to the manufacturer's protocol. The amplified libraries were purified using Ampure Beads and validated on Bioanalyzer High Sensitivity DNA Chips, and quantified by PicoGreen. All samples passed these QC tests and subsequently were entered into the MCC-Seq DNA methylation data production pipeline.

MCC-Seq DNA methylation profiling

The hybridization capture procedure of the amplified bisulfite converted library was performed with SeqCap Epi Enrichment System protocol (Roche NimbleGen) as described by the manufacturer. Using 1 μg of total input of library to hybridize with probes incubated at 47°C for 72 h. Washing and recovering of the captured library, as well as ligation-mediated PCR amplification and final purification, were carried out as recommended by the manufacturer. Quality, concentration and size distribution of the captured library was determined by Bioanalyzer DNA 1000 Chip. Each capture was sequenced on the Illumina HiSeq2500 system using 125 bp paired-end sequencing.

MCC-Seq methylation analysis

Clean sequences were first mapped to the human genome (build GRCh37) using Bismark (v0.10.1; parameters: –pe, –bowtie2, –directional, –unmapped) [5]. The following were removed: (i) adapter polluted reads: reads with > 5 bp adapter polluted base; reads at both ends if one end of the adapter is polluted for PE. (ii) low quality reads: reads with quality score (< 20) bases accounting for more than 15%; reads at both ends if one end with low quality score for PE. (iii) reads with N ratio > 5%; reads at both ends if one end with N ratio > 5% for PE (Supplementary Table 1). To avoid potential biases in downstream analyses, CpGs were further filtered as follows: CpGs not covered by at least five reads, CpGs not covered by at least two reads per strand.

We further selected CpGs sites that exhibited ≤ 20% methylation difference between strands. The minimum read coverage to call a methylation status for a base was set to 5. All off-target reads were removed. Methylation level at each site was determined by dividing the number of reads supporting methylation for that site by the total number of reads covering that site. CpGs were included in subsequent analysis if the number of sequence reads was 5 or greater. In some analyses, we also excluded sites at which the average sequence depth over all study individuals was below 5× coverage per site in the complete data set. CpGs were counted once per location combining both strands together. Additionally, for downstream analysis, repeat regions, sex, chromosomes and Mitochondrial chromosomes were excluded, and annotated using University of California Santa Cruz HG19. Differential Methylation regions (DMRs) were analyzed in R 3.1.0 using the methylKit package with data ≥ 10× [6]. Data visualization and analysis were performed using integrative genomics viewer (IGV), custom R and perl scripts [6–8].

To assess genome-wide DNA methylation patterns between different clinical subgroups, we calculated a DNA methylation indicator (DMI) for each sample (Supplementary Table 2). Differentially methylated genes for detected groups are determined by comparing the DNA-methylation profiling data of each sample of patients within the group vs. patients outside the group using the Student's *t* test. Genes are considered differentially methylated when DNA methylation levels differ with $P \leq 0.001$, after correcting for multiple testing using the Benjamini-Hochberg method (denoted as the false discovery rate, FDR) [9]. Pathway analysis is performed by using Molecular Signature Database, version 3.0 to detect enriched BioCarta pathways and, Kyoto Encyclopedia of Genes and Genomes (KEGG) pathways. Pathways and/or gene sets are considered statistically significant when the P value derived from the hypergeometric test is less than or equal to.05 after correcting for multiple testing using the FDR.

REFERENCES

1. Patel JP, Gonen M, Figueroa ME, Fernandez H, Sun Z, Racevskis J, Van Vlierberghe P, Dolgalev I, Thomas S, Aminova O, Huberman K, Cheng J, Viale A, et al. Prognostic relevance of integrated genetic profiling in acute myeloid leukemia. N Engl J Med. 2012; 366: 1079-89. https://doi.org/10.1056/NEJMoa1112304.
2. Mrozek K, Marcucci G, Nicolet D, Maharry KS, Becker H, Whitman SP, Metzeler KH, Schwind S, Wu YZ, Kohlschmidt J, Pettenati MJ, Heerema NA, Block AW, et al. Prognostic significance of the European LeukemiaNet standardized system for reporting cytogenetic and molecular alterations in adults with acute myeloid leukemia. J Clin Oncol. 2012; 30: 4515-23. https://doi.org/10.1200/JCO.2012.43.4738.
3. Dohner H, Estey E, Grimwade D, Amadori S, Appelbaum FR, Buchner T, Dombret H, Ebert BL, Fenaux P, Larson RA, Levine RL, Lo-Coco F, Naoe T, et al. Diagnosis and management of AML in adults: 2017 ELN recommendations from an international expert panel. Blood. 2017; 129: 424-47. https://doi.org/10.1182/blood-2016-08-733196.
4. Allum F, Shao X, Guenard F, Simon MM, Busche S, Caron M, Lambourne J, Lessard J, Tandre K, Hedman AK, Kwan T, Ge B; Multiple Tissue Human Expression Resource Consortium, et al. Characterization of functional methylomes by next-generation capture sequencing identifies novel disease-associated variants. Nat Commun. 2015; 6: 7211. https://doi.org/10.1038/ncomms8211.
5. Krueger F, Andrews SR. Bismark: a flexible aligner and methylation caller for Bisulfite-Seq applications. Bioinformatics. 2011; 27: 1571-2. https://doi.org/10.1093/bioinformatics/btr167.
6. Akalin A, Kormaksson M, Li S, Garrett-Bakelman FE, Figueroa ME, Melnick A, Mason CE. methylKit: a comprehensive R package for the analysis of genome-wide DNA methylation profiles. Genome Biol. 2012; 13: R87. https://doi.org/10.1186/gb-2012-13-10-r87.
7. Thorvaldsdottir H, Robinson JT, Mesirov JP. Integrative Genomics Viewer (IGV): high-performance genomics data visualization and exploration. Brief Bioinform. 2013; 14: 178-92. https://doi.org/10.1093/bib/bbs017.
8. Li S, Garrett-Bakelman FE, Akalin A, Zumbo P, Levine R, To BL, Lewis ID, Brown AL, D'Andrea RJ, Melnick A, Mason CE. An optimized algorithm for detecting and annotating regional differential methylation. BMC Bioinformatics. 2013; 14: S10. https://doi.org/10.1186/1471-2105-14-S5-S10.
9. Hochberg Y, Benjamini Y. More powerful procedures for multiple significance testing. Stat Med. 1990; 9: 811-8.
10. NimbleGen. www.nimblegen.com.
11. Blackwood EM, Kadonaga JT. Going the distance: a current view of enhancer action. Science. 1998; 281: 60-3. https://doi.org/10.1126/science.281.5373.60.
12. Pennacchio LA, Bickmore W, Dean A, Nobrega MA, Bejerano G. Enhancers: five essential questions. Nat Rev Genet. 2013; 14: 288-95. https://doi.org/10.1038/nrg3458.
13. Gao T, He B, Liu S, Zhu H, Tan K, Qian J. EnhancerAtlas: a resource for enhancer annotation and analysis in 105 human cell/tissue types. Bioinformatics. 2016; 32: 3543-51. https://doi.org/10.1093/bioinformatics/btw495.
14. Penalva LO, Sanchez L. RNA binding protein sex-lethal (Sxl) and control of Drosophila sex determination and dosage compensation. Microbiol Mol Biol Rev. 2003; 67: 343-59, table of contents.
15. Cenik C, Chua HN, Zhang H, Tarnawsky SP, Akef A, Derti A, Tasan M, Moore MJ, Palazzo AF, Roth FP. Genome analysis reveals interplay between 5'UTR introns and nuclear mRNA export for secretory and mitochondrial genes. PLoS Genet. 2011; 7: e1001366. https://doi.org/10.1371/journal.pgen.1001366.
16. Deaton AM, Bird A. CpG islands and the regulation of transcription. Genes Dev. 2011; 25: 1010-22. https://doi.org/10.1101/gad.2037511.
17. Jones PA. Functions of DNA methylation: islands, start sites, gene bodies and beyond. Nat Rev Genet. 2012; 13: 484-92. https://doi.org/10.1038/nrg3230.
18. Baylin SB, Chen WY. Aberrant gene silencing in tumor progression: implications for control of cancer. Cold Spring Harb Symp Quant Biol. 2005; 70: 427-33. https://doi.org/10.1101/sqb.2005.70.010.
19. Brenet F, Moh M, Funk P, Feierstein E, Viale AJ, Socci ND, Scandura JM. DNA methylation of the first exon is tightly linked to transcriptional silencing. PLoS One. 2011; 6: e14524. https://doi.org/10.1371/journal.pone.0014524.
20. Clermont PL, Parolia A, Liu HH, Helgason CD. DNA methylation at enhancer regions: novel avenues for epigenetic biomarker development. Front Biosci (Landmark Ed). 2016; 21: 430-46. https://doi.org/10.2741/4399.
21. Heyn H, Vidal E, Ferreira HJ, Vizoso M, Sayols S, Gomez A, Moran S, Boque-Sastre R, Guil S, Martinez-Cardus A, Lin CY, Royo R, Sanchez-Mut JV, et al. Epigenomic analysis detects aberrant super-enhancer DNA methylation in human cancer. Genome Biol. 2016; 17: 11. https://doi.org/10.1186/s13059-016-0879-2.

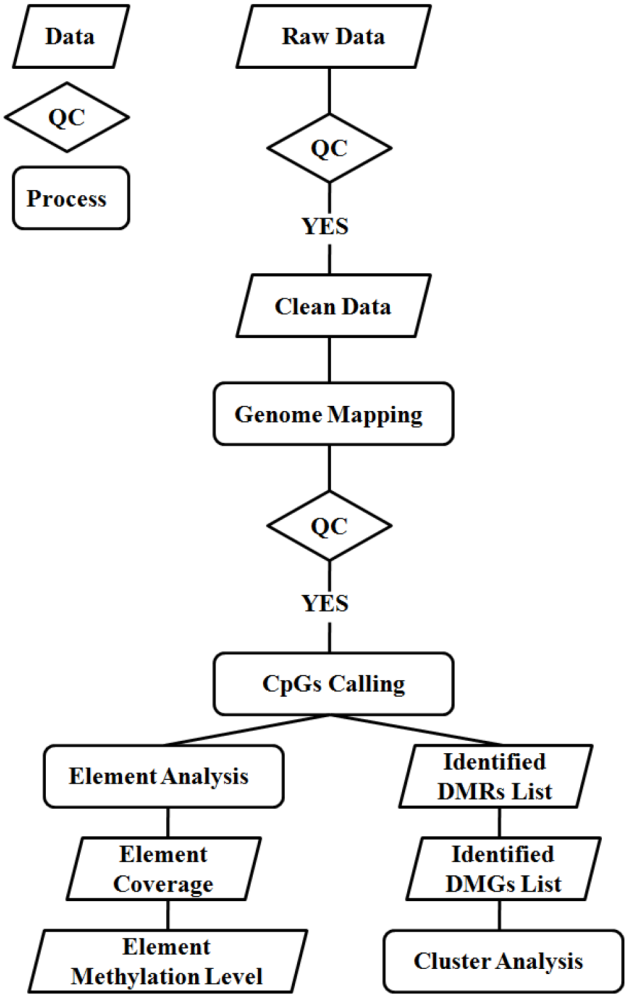

Supplementary Figure 1: Diagram of MCC-Seq methylation analysis workflow. Parallelograms, Data; Diamonds, quality control (QC); Rectangles, Process.

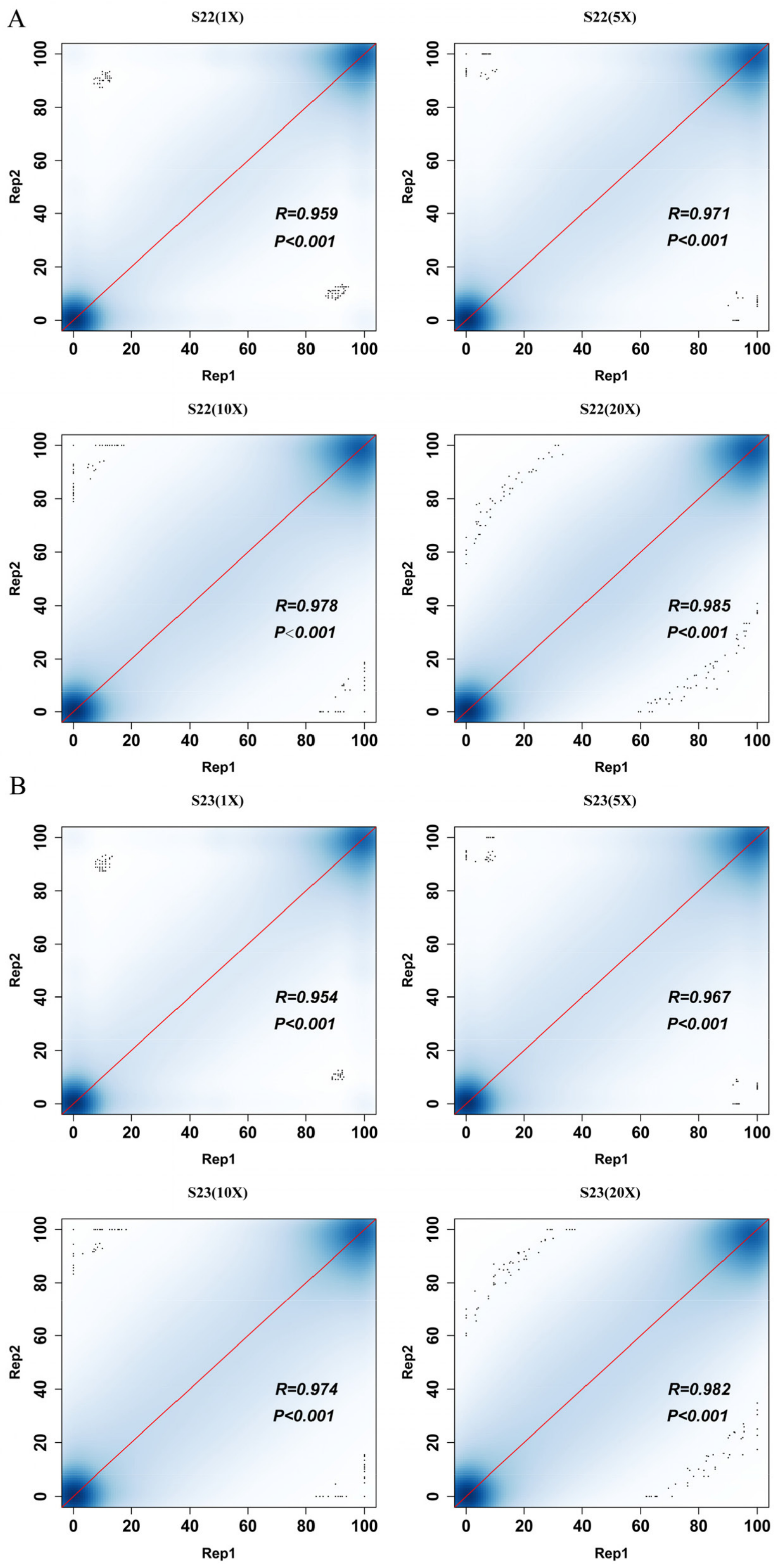

Supplementary Figure 2: Technical replication of MCC-Seq methylation calls. Correlation between technical replicates from DNA samples (S22-Rep1 and S22-Rep2; S23-Rep1 and S23-Rep2) derived from replicate BM samples of C22 **(A)** and C 23 **(B)**. R is the Pearson's correlation coefficient.

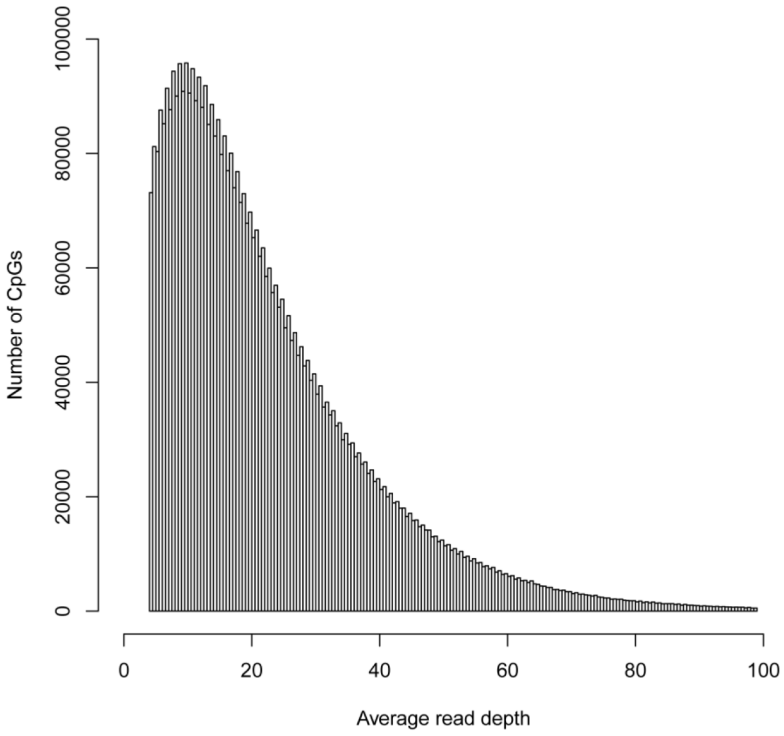

Supplementary Figure 3: Distributions of sequence coverage at included CpG sites. Distributions of the average read depth across the 37 individuals of included CpG sites when considering all CpG sites with ≥ 5× and ≤ 100×.

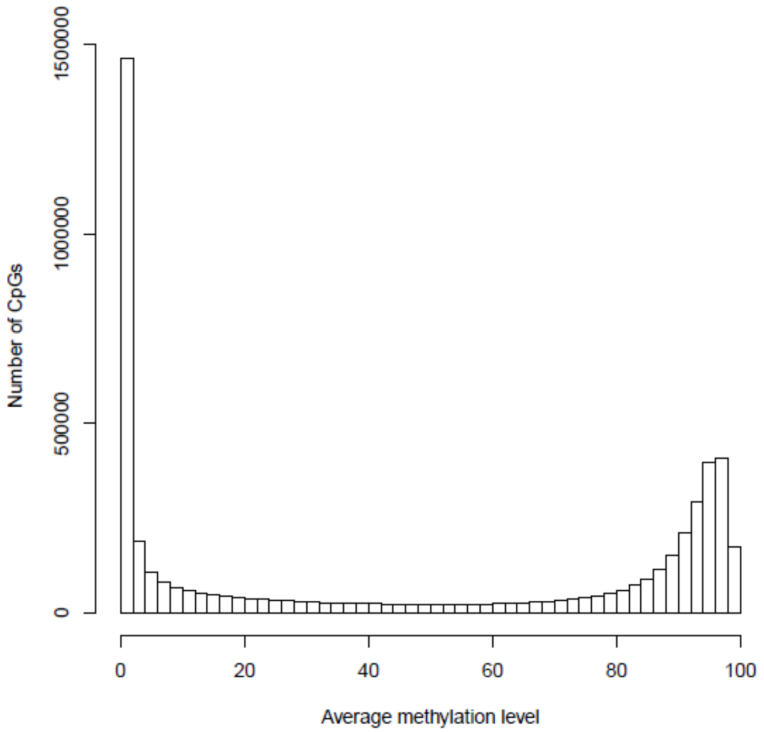

Supplementary Figure 4: Average methylation levels of CpGs captured with MCC-Seq. The figure shows the average methylation level (%, x-axis) for 21 de novo AML samples of captured CpGs at the depth of 10×.

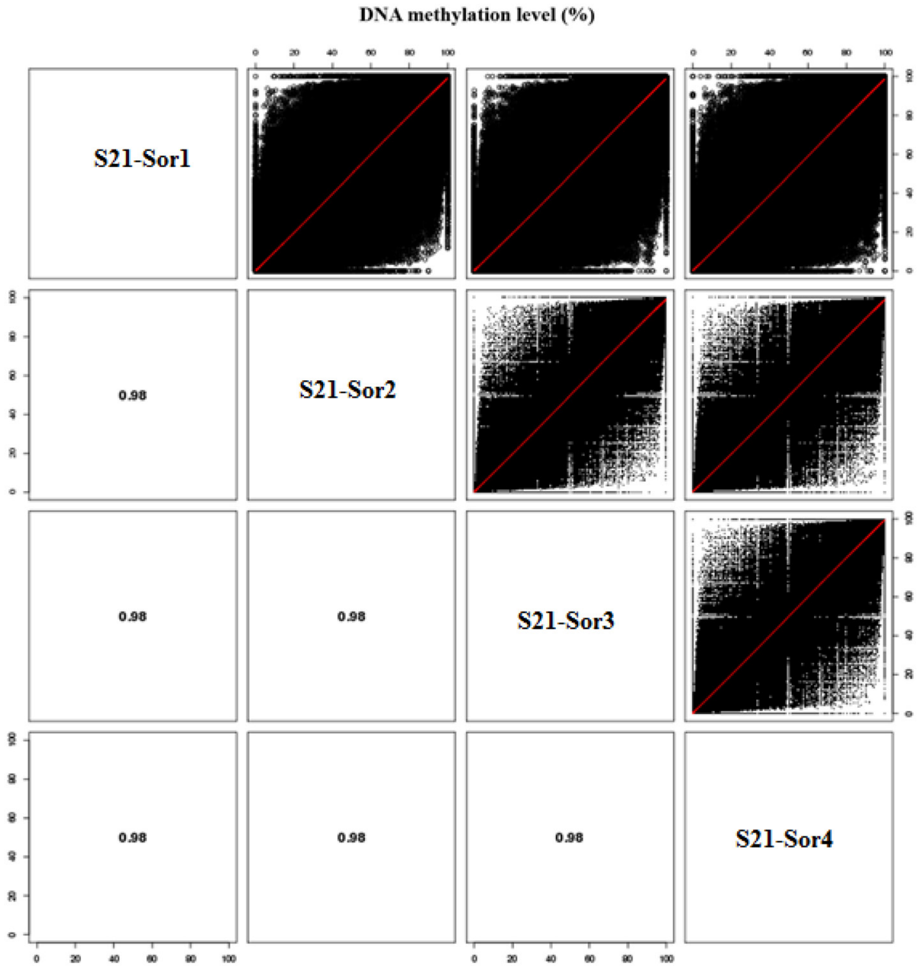

Supplementary Figure 5: Correlation between DNA methylation level and percentage blast of specimens. The figure shows that DMI was not affected by the percentage of blast. R is the Pearson's correlation coefficient.

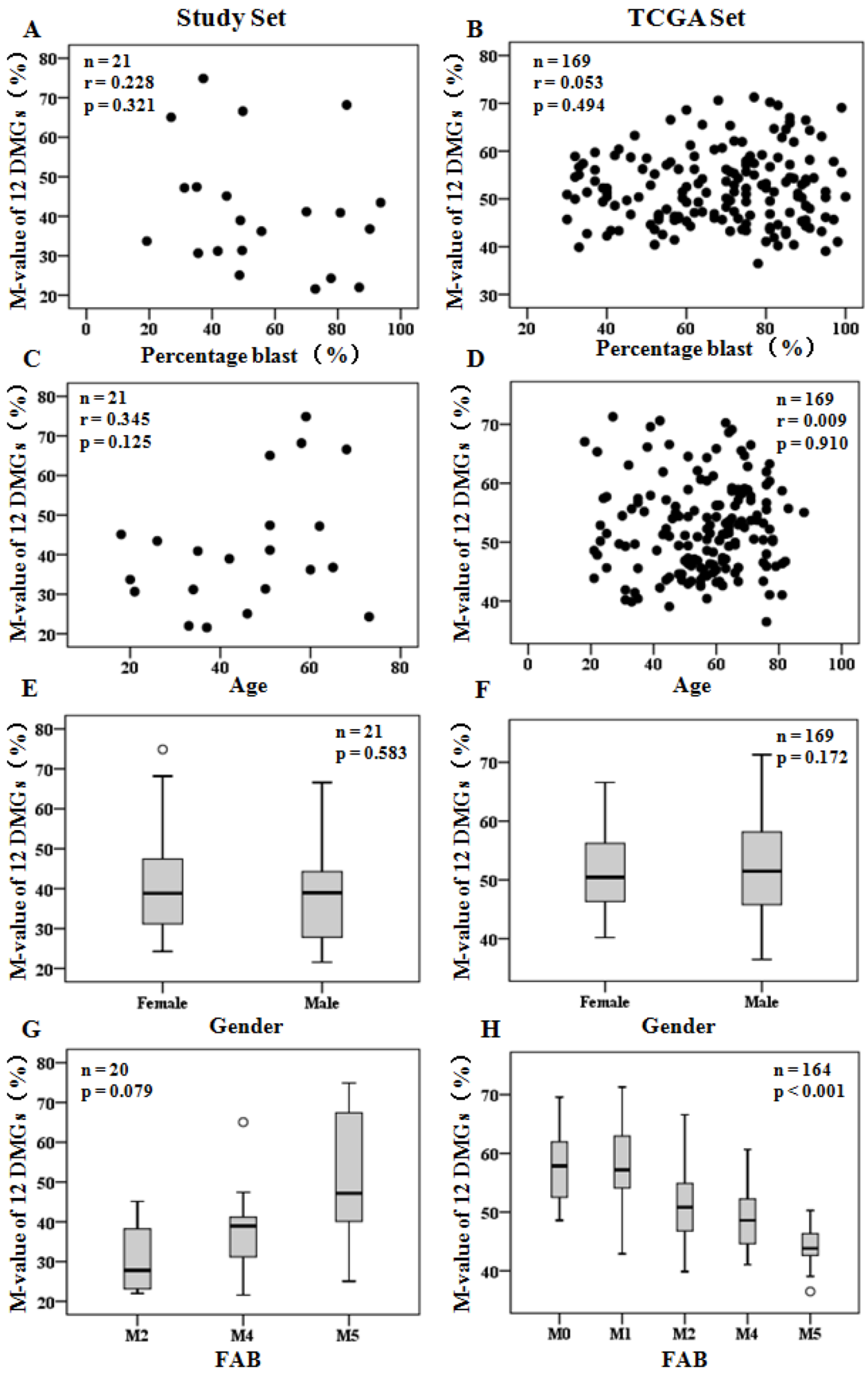

Supplementary Figure 6: Correlation between M-value and clinical characteristics in study set and TCGA set. **(A-D)** Scatter plots of M-value (%) compared to patient percentage blast of specimens and ages. **(E-H)** Box plots of M-value (%) grouped by patient gender (E-F), and FAB disease classification (G-H). Pearson correlation was used to calculate r and Student's *t* test or one-way ANOVA was used for mean tests.

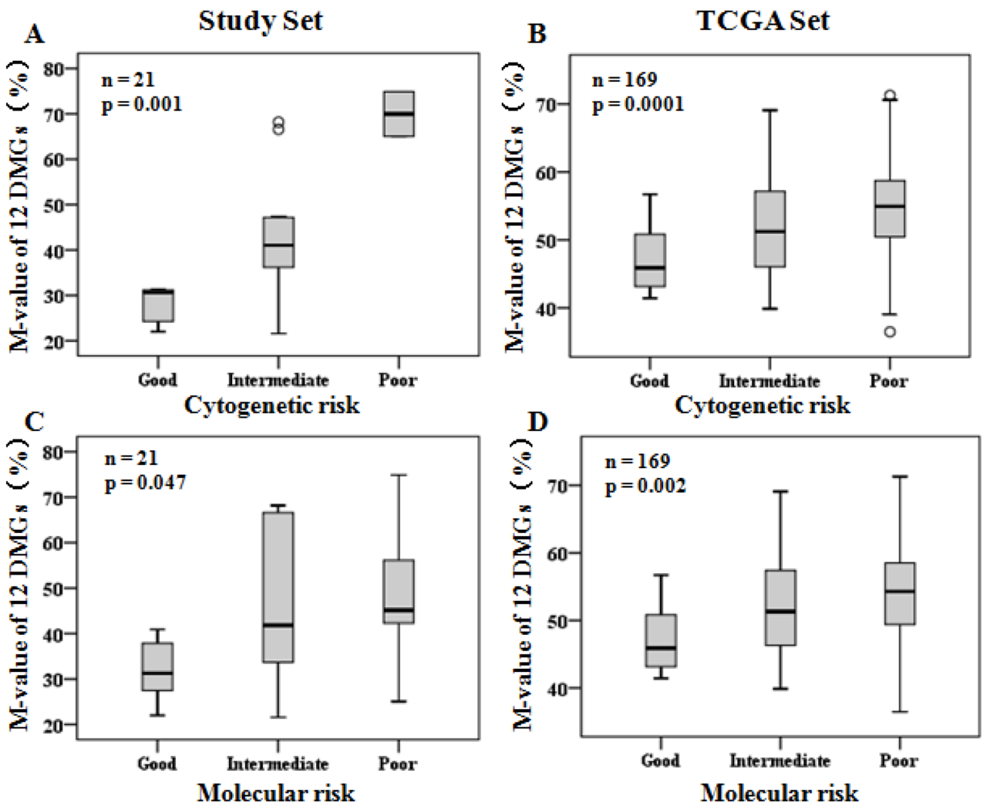

Supplementary Figure 7: Correlation between M-value and genetic characteristics in study set and TCGA set. (A-B) Box plots of M-value (%) grouped by cytogenetic risk status. **(C-D)** Box plots of M-value (%) grouped by molecular risk status. One-way ANOVA was used for mean tests.

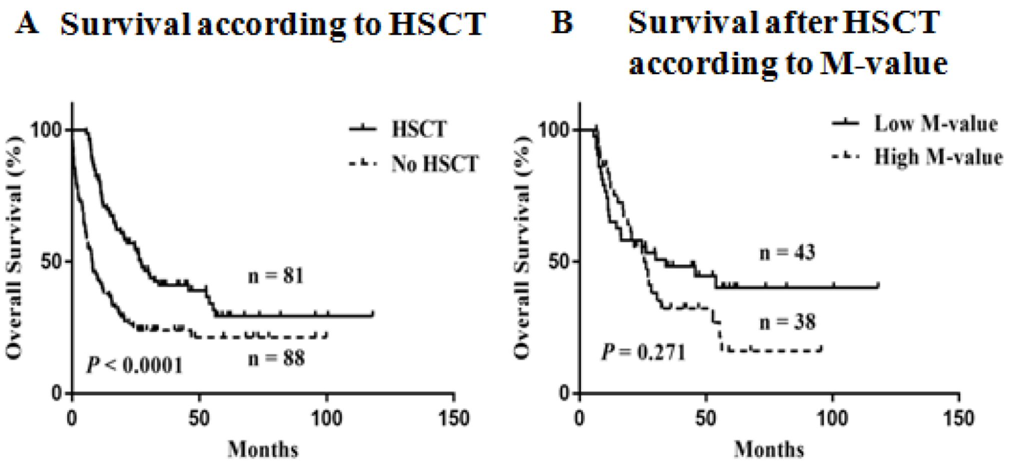

Supplementary Figure 8: Correlation between HSCT or M-value and survival. **(A)** showes the rate of overall survival among 169 patients according to whether they had undergone HSCT. **(B)** showes the rate of survival among patients (n = 81) who underwent HSCT according to M-value. P values were calculated with the use of log-rank analysis.

Supplementary Table 1: The sequence quality control statistics including coverage, cleaned and mapped reads statistics

See Supplementary File 1

Supplementary Table 2: Glossaries in this study

**Glossaries**	
CpG islands	Minimum criteria as defined in UCSC Genome Browser: 200 bp; 50% GC content; CpG observed-overexpected ratio of 0.6.
CpG sits (CpGs)	The CpG sites or CG sites are regions of DNA where a cytosine nucleotide is followed by a guanine nucleotide in the linear sequence of bases along its 5' → 3' direction.
DNA methylation	This generally refers to the addition of a methyl group to the 5-carbon of the pyrimidine ring of cytosine, usually pertaining to cytosines that precede a guanine residue in DNA (a CpG dinucleotide motif). DNA methylation of CpG-rich regions (CpG islands) is often associated with repression of nearby genes.
Differentially Methylated Regions (DMRs)	Regions within genomic features that show a statistically significant difference in methylation levels: 1) at least including 5 CpGs; 2) no less than 3 differentially methylated CpGs [6, 8].
Differentially Methylated Genes (DMGs)	Genes annotated referring to the DMRs in functional elements, mainly enriched in the promoter and enhancer regions [10].
DNA methylation indicator (DMI)	The DNA methylation level for each locus/regions was presented as DMI: DMI = Number of reads supporting methylation / the total number of reads converting x 100% [6, 8].
Differentially Methylated CpGs	CpGs within genomic features that show a statistically significant difference in methylation levels: 1) differentially DMI no less than 20%; 2) with a q value (P value corrected for multiple hypothesis testing) < 0.05 [6, 8, 9].
RefSeq genes	The UCSC Genome Browser definition of RefSeq was used; RefSeq genes were defined as Human protein-coding and non–protein-coding genes collected by NCBI RNA reference sequences.
RefSeq gene promoters	1kp upstream and 500 bp downstream of transcriptional start site (TSS) of RefSeq genes.
RefSeq gene Exons	The exons of RefSeq gene transcripts as annotated in the RefSeq gene track on UCSC Genome Browser.
RefSeq gene Introns	The Introns of RefSeq gene transcripts as annotated in the RefSeq gene track on UCSC Genome Browser.
RefSeq gene Exon 1	The first exon of RefSeq gene transcripts as annotated in the RefSeq gene track on UCSC Genome Browser.
Enhancer	In genetics, an enhancer is a short (50-1500 bp) region of DNA that can be bound by proteins (activators) to increase the likelihood that transcription of a particular gene will occur [11, 12]. In this study, the enhancer annotation and analysis were based on dataset of CD34+ cells of Blood from http://enhanceratlas.org [13].
5'untranslated region (5'UTR)	The 5'UTR (also known as a Leader Sequence or Leader RNA) is the region of an mRNA that is directly upstream from the initiation codon. This region is important for the regulation of translation of a transcript [14, 15].

NOTE. CpG islands: their association with gene transcription is recently reported by Deaton and Bird [16]. RefSeq genes: methylation level of genomic sequences (including exons, introns, and enhancers) used as reference standards for well-characterized genes may affect gene transcription [17]. RefSeq gene promoters: promoter methylation may impact expression in well characterized RefSeq genes [18]. RefSeq gene exon 1: The first exon methylation may impact expression of well characterized RefSeq genes [19]. Enhancer: evidence demonstrates that enhancer sequences also undergo extensive differential methylation in cancer cells [20, 21]. UCSC, University of California, Santa Cruz; TSS, transcription start site; NCBI, National Center for Biotechnology Information.

Supplementary Table 3: Differentially methylated genes in promoters compared among cytogenetic and molecular prognostic stratifications

See Supplementary File 2

Supplementary Table 4: Correlation between M-value and AML induction remission in study set

	Low M-value (n = 11)	High M-value (n = 10)	Total (n = 21)
**7+3**			
CR	4	2	6
NR	0	3	3
CR rate	100% (4/4)	40.0% (2/5)	66.7% (6/9)
**DCAG**			
CR	6	2	8
NR	1	3	4
CR rate	85.7% (6/7)	40.0% (2/5)	66.7% (8/12)
**Total**	90.9% (10/11)	40.0% (4/10)	66.7% (14/21)

"7+3", standard induction regimens based on a backbone of cytarabine plus an anthracycline, details according to the NCCN guideline for AML; DCAG, decitabine 20mg/m^2 d1-5, cytarabine 10mg/m^2 q12h d1-5, aclarubicin 20mg d1, 3, 5, granulocyte colony-stimulating factor (G-CSF) 300µg/d from d0 until recovery from neutropenia.
CR, complete remission; NR, no response.

Supplementary Table 5: Patient and disease characteristics by optimal M-value for the study set and TCGA set

	Study set (n = 21)			TCGA set (n = 169)		
	**Low M-value (n = 11) n, %**	**High M-value (n = 10) n, %**	***P* value**	**Low M-value (n = 85) n, %**	**High M-value (n = 84) n, %**	***P* value**
Age			0.670			0.774
< 50 years	6 (54.5)	4 (40.0)		26 (30.6)	24 (28.6)	
≥ 50 years	5 (45.5)	6 (60.0)		59 (69.4)	60 (71.4)	
Gender			0.183			0.933
Female	9 (81.8)	5 (50.0)		39 (45.9)	38 (45.2)	
Male	2 (18.2)	5 (50.0)		46 (54.1)	46 (54.8)	
WBC (x 10^9/L)			0.476			0.210
< 100	9 (81.8)	10 (100.0)		74 (87.1)	78 (92.9)	
≥ 100	2 (18.2)	0 (0)		11 (12.9)	6 (7.1)	
Cytogenetics risk			0.009			0.001
Good	5 (45.5)	0 (0)		16 (18.8)	3 (3.6)	
Intermediate	6 (54.5)	8 (80.0)		56 (65.9)	52 (61.9)	
Poor	0 (0)	2 (20.0)		13 (15.3)	29 (34.5)	
Molecular risk			0.001			0.001
Good	8 (72.7)	0 (0)		16 (18.8)	3 (3.6)	
Intermediate	2 (18.2)	4 (40.0)		52 (61.2)	49 (58.3)	
Poor	1 (9.1)	6 (60.0)		17 (20.0)	32 (38.1)	
HSCT			0.635			0.486
Yes	4 (36.4)	2 (20.0)		43 (50.6)	38 (45.2)	
No	7 (63.6)	8 (80.0)		42 (49.4)	46 (54.8)	

WBC, white blood cells; HSCT, hematopoietic stem cell transplantation.

Supplementary Table 6: Clinical information of the de novo AML patients and all samples

See Supplementary File 3

Supplementary Table 7: Clinical information of the TCGA set used in this study (n =169)

See Supplementary File 4
